# The Impact of Pharmacist-Managed Service on Warfarin Therapy in Patients after Mechanical Valve Replacement

**DOI:** 10.1155/2022/1617135

**Published:** 2022-03-17

**Authors:** Chia-Wei Wu, Chien-Chih Wu, Chien-Hao Chen, Shin-Yi Lin, Ron-Bin Hsu, Chih-Fen Huang

**Affiliations:** ^1^Department of Pharmacy, National Taiwan University Hospital, Taipei, Taiwan; ^2^School of Pharmacy, College of Medicine, National Taiwan University, Taipei, Taiwan; ^3^Department of Surgery, National Taiwan University Hospital, Taipei, Taiwan

## Abstract

**Objective:**

To evaluate the impact of pharmacist interventions on international normalized ratio (INR) control during the warfarin initiation phase after mechanical valve replacement.

**Methods:**

This was a retrospective cohort study conducted in a cardiovascular surgery ward in a tertiary hospital from August 1, 2015, to July 31, 2019. Patients aged ≥20 years who were admitted for mechanical valve replacement were enrolled in this study and further classified into conventional and pharmacist-managed warfarin therapy (PMWT) groups. All participants were prospectively followed up until the first outpatient appointment after valve replacement. The effectiveness outcomes were time in therapeutic range (TTR), time to therapeutic INR, number of patients with therapeutic INR at discharge and at first outpatient appointment, and length of hospital stay. The safety outcome was the number of patients with any supratherapeutic INR during the hospital stay. Multivariate logistic regression analyses were also used to determine the predictors of a therapeutic INR at discharge or with any supratherapeutic INR during admission.

**Results:**

A total of 39 and 33 patients were enrolled in the conventional and PMWT groups, respectively. At discharge, 18 patients (46.2%) in the conventional group and 24 patients (72.7%) in the PMWT group had achieved the therapeutic INR (*P*=0.023). Compared to the conventional group, fewer patients in the PMWT group had supratherapeutic INR during hospital stay (35.9% vs. 9.0%, *P*=0.008). No significant differences were found in TTR, time to therapeutic INR, number of patients with therapeutic INR at return appointment, and length of stay between the study groups. In the multivariate regression analyses, PMWT predicted achieving therapeutic INR at discharge (odds ratio (OR) and 95% confidence interval (CI), 3.14 [1.08–9.14]) and was inversely associated with supratherapeutic INRs during admission (OR = 0.21 [0.05–0.82]).

**Conclusions:**

Among patients admitted for mechanical valve replacement, the implementation of PMWT was associated with optimal therapeutic INR at discharge and no supratherapeutic INR during admission. Therefore, pharmacist participation is essential for improving the quality of warfarin therapy.

## 1. Background

Warfarin, a vitamin K antagonist and oral anticoagulant, is recommended for all patients after mechanical valve replacement to prevent valve thrombosis and systemic embolic events [1, 2]. To ensure an anticoagulation response and reduce the bleeding risk, it is important to maintain the international normalized ratio (INR) within the therapeutic range. Due to the significant impact of drugs, comorbidities, diet, and genetic polymorphisms on warfarin pharmacokinetics and pharmacodynamics, frequent INR monitoring is required for dosage adjustment, especially after open-heart surgery. The causes of increased warfarin sensitivity after cardiac surgery include hypoalbuminemia and reduced clotting factor concentrations after cardiopulmonary bypass, reduced oral intake and physical activity, and drug interactions with antiplatelet agents, amiodarone, nonsteroidal anti-inflammatory drugs (NSAIDs), and antibiotics [3, 4]. In ambulatory care, acute settings, or inpatient settings, previous studies have demonstrated that pharmacist-managed warfarin therapy (PMWT) increased the percentage of time for INR in the therapeutic range and patient satisfaction, improved clinical outcomes, and was cost-saving and cost-effective compared to conventional care [5–10]. However, few investigations have focused specifically on patients who have undergone cardiac surgery. The aim of this study was to evaluate the impact of PMWT on INR control during the warfarin initiation phase in patients after mechanical valve replacement.

## 2. Method

### 2.1. Medical Setting and Patients

This retrospective cohort study was conducted in a 30-bed cardiovascular surgery ward in a tertiary hospital, which is a tertiary medical center. Since August 1, 2017, a clinical pharmacist participated in the medical team of this cardiovascular surgery ward and provided several pharmacy services, including the PMWT.

Patients over 20 years of age who were admitted for mechanical valve replacement with newly started warfarin therapy between August 1, 2015, and July 31, 2019, were enrolled in this study. Patients enrolled before July 31, 2017, which was before the date of the implementation of PMWT, were classified into the conventional group. Patients enrolled between August 1, 2017, and July 31, 2019 were defined as the PMWT group. Patients were excluded if they received warfarin within 1 year before surgery and had embolic or hemorrhagic stroke caused by infective endocarditis before surgery. This study was approved by the institutional review board and ethics committee (No. 202004047RINC).

### 2.2. Target of Anticoagulant Therapy

Warfarin was prescribed for patients after mechanical valve replacement based on the recommendation of the European Society of Cardiology. Antiplatelet agents should be considered in cases of concomitant atherosclerotic disease or thromboembolism, despite an adequate INR [1, 11]. Therapeutic INR was defined as 1.5–3.0 and 1.8–3.0 for patients who received aortic valve replacement (AVR) and mitral valve replacement (MVR) or double valve replacement (DVR), respectively [12]. The INRs were checked at least twice weekly during the hospital stay and at the first appointment after discharge.

### 2.3. PMWT and Conventional Care

Before the implementation of PMWT, the initial dose and dose adjustment of warfarin and INR monitoring were managed by the attending physicians based on their clinical experience. Patients received warfarin education from a nurse or pharmacist with an instruction leaflet.

The clinical pharmacist, who had 3 years of experience in pharmaceutical care at a pharmacist-managed anticoagulation clinic and cardiovascular surgery intensive care unit, was in charge of PMWT at the study site since August 1, 2017. Every Monday to Friday from 8 am to 5 pm, the clinical pharmacist participated in the ward check rounds with healthcare practitioners to provide medication therapy management for all patients admitted to the cardiovascular surgery ward. The processes of PMWT included (1) providing suggestions for initial dose and dose adjustment of warfarin [12]; (2) developing a monitoring plan for INR and any adverse drug reactions; (3) documenting and managing the drug-drug and drug-food interactions in patients; (4) identifying causes of a supratherapeutic INR and providing suggestions for the management of excessive anticoagulation; and (5) providing detailed education to individual patients and their caregivers. The PMWT provided by the clinical pharmacist was guided by the warfarin dosing protocol in general (Table S1) but was tailored to each individual by evaluating factors that influence warfarin sensitivity, such as disease status, comorbidities, comedications, and dietary habits. All of the advice from the clinical pharmacist was documented in the electronic medical records. The warfarin education provided by the clinical pharmacist involved 30–60 minutes of bedside visits. The educational content included the importance and precautions of warfarin, practical, and manageable plans for consistent vitamin K diet, advice for concomitant complementary or herbal medicines, and a personalized instruction leaflet. The PMWT was mainly implemented in inpatient services. After discharge, the patients could contact the medical team if they had any problems, but the pharmacist did not preemptively monitor them after discharge.

### 2.4. Clinical Data Acquisition

Data on baseline demographics, surgery records, warfarin dose, and INRs during hospital stay and at the first appointment after discharge were collected from the electronic medical records of the patients. Drugs frequently used concurrently with warfarin, documented drug interactions with warfarin of severity higher than moderate, and evidence higher than good were also documented, including amiodarone, antiplatelet agents, benzbromarone, fluoroquinolones, and azole antifungal agents. The severity and evidence of drug interactions with warfarin were based on the Micromedex, drug interaction database [13].

### 2.5. Outcome of Interest

The effectiveness outcomes included (1) time in therapeutic range (TTR), calculated by the Rosendaal method [14], defined as the percentage of days for INR in the therapeutic range until hospital discharge (therapeutic INR was 1.5–3.0 for AVR and 1.8–3.0 for MVR or DVR); (2) time to therapeutic range, defined as days to the first INR in the therapeutic range after warfarin initiation; (3) number of patients with therapeutic INR at discharge; (4) number of patients with therapeutic INR at first return appointment after discharge; and (5) length of hospital stay after warfarin initiation. The safety outcome was the number of patients with any supratherapeutic INR during the hospital stay.

All participants were observed from the date of valve surgery until the first outpatient appointment. Thromboembolic events and major bleeding during the observation period were also recorded, including venous thromboembolism, ischemic stroke, and valve thrombosis. Major bleeding was defined according to the International Society on Thrombosis and Hemostasis (ISTH) criteria to fulfill one or more of the following [15, 16]: (1) fatal bleeding; (2) symptomatic bleeding in a critical area or organ; and (3) bleeding causing a fall in hemoglobin level of 2 g/dL or more, or leading to transfusion of two or more units of whole blood or red cells.

### 2.6. Statistical Analyses

Descriptive analysis was used to present the average and standard deviations. Comparisons of all baseline characteristics and outcome measures between study groups were conducted using the chi-square test or Fisher's exact test for categorical variables and the student's *t* test or Mann–Whitney *U* test for continuous variables, as appropriate. Multivariate logistic regression analyses were performed to determine the predictors of a therapeutic INR at discharge or with any supratherapeutic INR during admission. Statistical significance was set at *P* < 0.05. All statistical analyses were performed using the STAT v.14.0 software (StataCorp., 2015. Stata Statistical Software: Release 14. College Station, TX, StataCorp LP)

## 3. Results

**Figure 1:**
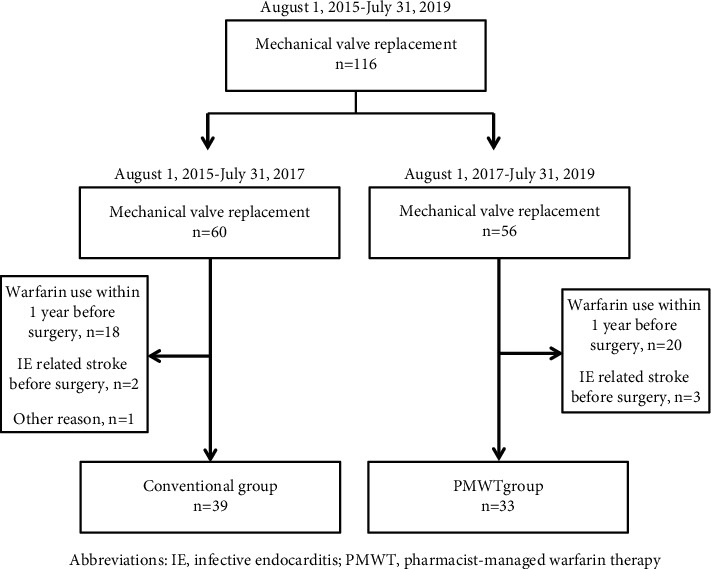
The study enrollment.

Between August 1, 2015, and July 31, 2019, a total of 116 patients were admitted for mechanical valve replacement. Among them, 38 patients were excluded because they had used warfarin before study enrollment, 5 patients were excluded due to infective endocarditis-related stroke before surgery, and 1 patient was excluded due to prolonged hospitalization over 1 year. The remaining 72 patients were enrolled, including 39 patients in the conventional group and 33 in the PMWT group, as depicted in Figure 1. The average follow-up duration was 30.54 ± 19.89 days for the conventional group and 28.12 ± 13.29 days for the PMWT group.

Comparisons between the conventional and PMWT groups are presented in Table 1. Compared with the PMWT group, participants in the conventional group had a higher mean left ventricular ejection fraction (65.0 ± 11.6% versus 56.7 ± 15.7%, *P*=0.033). Other demographic characteristics and comorbid conditions were similar between the two groups.

The effectiveness outcomes are summarized in Table 2. More number of patients in the PMWT group reached therapeutic INR at discharge compared to the conventional group (24 patients [72.7%] versus 18 patients [46.2%], *P*=0.023, respectively). Nevertheless, no significant differences were observed in the time to therapeutic range, TTR, number of patients with therapeutic INR at return appointment, and length of stay between the study groups. The safety outcomes are listed in Table 2. Compared with the conventional group, fewer participants in the PMWT group had supratherapeutic INR during hospital stay (3 patients [9.1%] versus 14 patients [35.9%], *P*=0.008, respectively). In addition, most patients with supratherapeutic INR had an INR between 3 and 5, not at a higher level. Regarding clinical outcomes, no thromboembolic events occurred during the observation period, either in the conventional or PMWT groups. In contrast, one major bleeding event (gastrointestinal bleeding) occurred in the conventional group, but none were observed in the PMWT group.

In multivariate logistic regression analysis, after adjusting age, sex, left ventricular ejection fraction, and renal and liver function, PMWT remained significant factors to predict therapeutic INR at discharge (Odds ratio (OR) = 3.33 [1.03–10.76], *P*=0.045), and as a protective factor for the occurrence of supratherapeutic INR during hospital stay (OR = 0.19 [0.04–0.84], *P*=0.029), as displayed in Table 3.

## 4. Discussion

To the best of our knowledge, this is the first study conducted in Asians to investigate the impact of PMWT on the quality of warfarin therapy after mechanical valve replacement. Our data showed that PMWT was associated with achieving a therapeutic INR at discharge and having no supratherapeutic INR during admission.

Several previous studies have demonstrated the benefits of PMWT in inpatient care. However, patient populations, type of pharmacist interventions, and outcome measurements varied across studies [17–21]. Further, none of the investigations focused specifically on patients admitted for mechanical valve replacement, a population with clear indications for long-term warfarin therapy. One retrospective study compared PMWT to physician care among patients admitted for cardiac valve surgery. The results showed that a pharmacist-managed dosing nomogram reduced the incidence of INR > 4 but did not change the TTR, proportion of patients with stabilized INR before discharge, and major bleeding events [17]. Similarly, another study compared the protocol-guided anticoagulation management service to conventional care in a cardiac surgery ward (80% of patients underwent valve surgery), which showed a reduced proportion of supratherapeutic INR but comparable incidence of bleeding [21]. In contrast to merely depending on protocols, our facility implemented PMWT in the cardiac surgery ward by adding a clinical pharmacist to the medical team. The warfarin dosing protocol was adjusted individually by evaluating the factors that could influence warfarin sensitivity. Our data showed that PMWT not only reduced the occurrence of supratherapeutic INR during admission but also helped reach therapeutic INR upon discharge. In addition, none of the participants in the PMWT group were discharged with a supratherapeutic INR, which may be linked to the bleeding risk associated with warfarin therapy.

In our present study, in the PMWT group, the time to therapeutic INR was numerically lower, and the TTR was numerically higher than that in the conventional group. However, this difference did not reach the level of significance. According to the pharmacological properties of warfarin, a full anticoagulation effect occurs 5–10 days after initiation or dose adjustment [3, 17]. Considering that the duration of hospital stay was only around 2 weeks, it may be reasonable for some nonsignificant differences in time to therapeutic INR and TTR between the study groups. Some studies indicated that PMWT significantly reduced the mean length of hospital stays by 2–3 days [18–21]. In our study, the length of hospital stay was similar between the two groups. Factors influencing the length of hospital stay were multifocal, including the etiologies of valve disease, methods of valve surgery, scheduled surgery or emergent operation, and the occurrence of complications after surgery.

Our study did have some limitations that need to be interpreted cautiously. First, due to the strict inclusion criteria, the number of participants was small in our investigation. In addition, only one bleeding event occurred during the observation period. Therefore, we were not able to assess the impact of PWMT on the clinical outcomes. Nevertheless, the main purpose of our investigation was to analyze the impact of PMWT on INR control during the warfarin initiation phase. Continuous implementation of PMWT in an outpatient setting is essential and our future direction of investigation. Second, this is a prepost design study, and not all changes over time could be assessed between the study groups, such as techniques for valve surgery and quality of medical care. Finally, this was a single-center study; however, as the PMWT varies across different medical settings, future larger-scale and multicenter investigations are necessary to confirm these findings.

## 5. Conclusions

Among the patients admitted for mechanical valve replacement, the implementation of PMWT was significantly associated with achieving a therapeutic INR at discharge and avoided supratherapeutic INR during hospital stay. Therefore, pharmacist participation is essential for improving the quality of warfarin therapy.

## Figures and Tables

**Table 1:** Baseline characteristics of study groups.

	Conventional group (*n* = 39)	PMWT group (*n* = 33)	*P* value
Patient demographics			
Male	26 (66.7)	27 (81.8)	0.146
Age (years)	47.4 ± 13.6	47.6 ± 13.4	0.914
Body weight (kg)	67.1 ± 16.2	68.9 ± 16.5	0.484
LVEF (%)	65.0 ± 11.6	56.7 ± 15.7	0.033^*∗*^
Serum creatinine >1.5 mg/dL	4 (10.3)	1 (3.0)	0.366
Total bilirubin >2.0 mg/dL	2 (5.1)	6 (18.2)	0.131
ALT >3 ULN	1 (2.6)	1 (3.0)	1.000
Diagnoses			
Aortic valve regurgitation	20 (51.3)	18 (54.6)	0.782
Aortic valve stenosis	11 (28.2)	6 (18.2)	0.318
Mitral valve regurgitation	17 (43.6)	9 (27.3)	0.151
Mitral valve stenosis	5 (12.8)	2 (6.1)	0.442
Infective endocarditis	7 (18.0)	6 (18.2)	0.980
Types of operation			
Primary cardiac surgery	32 (82.1)	28 (84.9)	0.751
Emergent surgery	10 (25.6)	3 (9.1)	0.069
Operation methods			
Bentall procedure	7 (18.0)	10 (30.3)	0.219
Aortic valve replacement	14 (35.9)	13 (39.4)	0.760
Mitral valve replacement	12 (30.8)	9 (27.3)	0.745
Double valve replacement	6 (15.4)	1 (3.0)	0.116
Comorbidities			
Hypertension	17 (43.6)	13 (39.4)	0.719
Vascular disease	9 (23.1)	7 (21.2)	0.850
Atrial fibrillation	6 (15.4)	5 (15.2)	0.978
Diabetes mellitus	6 (15.4)	3 (9.1)	0.494
History of ischemic stroke	1 (2.6)	1 (3.0)	1.000
End-stage renal disease	2 (5.1)	1 (3.0)	1.000
Drug-drug interactions			
Any DDIs	18 (46.2)	18 (54.6)	0.478
Amiodarone	11 (28.2)	8 (24.2)	0.704
Antiplatelet agents	7 (18.0)	10 (30.3)	0.219
NSAIDs	2 (5.1)	7 (21.2)	0.070

Data are represented as the number of patients (%) or mean ± SD. ^*∗*^Statistically significant (*P* < 0.05). ALT, alanine aminotransferase; DDIs, drug-drug interactions; LVEF, left ventricular ejection fraction; NSAIDs, nonsteroidal anti-inflammatory drugs; SD, standard deviation; ULN, upper limit of normal; PMWT, pharmacist-managed warfarin therapy.

**Table 2:** Effectiveness and safety outcomes of study groups.

Outcomes	Conventional group (*n* = 39)	PMWT group (*n* = 33)	*P* value
Effectiveness outcomes			
Time in therapeutic range (%)	37.1 ± 26.6	44.0 ± 32.4	0.327
Time to therapeutic range (days)	7.6 ± 5.9	6.2 ± 3.9	0.304
Therapeutic INR at discharge	18 (46.2)	24 (72.7)	0.023^*∗*^
Subtherapeutic INR at discharge	18 (46.2)	9 (27.3)	0.099
Supratherapeutic INR at discharge	3 (7.7)	0 (0.0)	0.245
Therapeutic INR at first return appointment^†^	22 (62.9)	22 (71.0)	0.485
Length of stays (days)	18.9 ± (14.7)	16.4 ± (9.5)	0.789
Safety outcome			
Supratherapeutic INR during hospital stay	14 (35.9)	3 (9.1)	0.008^*∗*^
Distribution of supratherapeutic INR			
3 < INR ≤ 5	12 (85.7)	1 (33.3)	0.002^*∗*^
5 < INR ≤ 9	2 (14.3)	1 (33.3)	1.000
INR ≥ 9	0 (0)	1 (33.3)	0.458

Data are represented as the number of patients (%) or mean ± SD. ^*∗*^Statistically significant (*P* < 0.05). INR, international normalized ratio; PMWT, pharmacist-managed warfarin therapy; SD, standard deviation. ^†^A total of 4 patients in the conventional group and 2 patients in the PMWT group did not return to the first outpatient appointment after discharge.

**Table 3:** Multivariate logistic regression models for factors associated with the outcomes of interests.

Factors	Therapeutic INR at discharge		Supratherapeutic INR during hospital stay	
	OR (95% CI)	*P* value	OR (95% CI)	*P* value
PMWT	3.33 (1.03–10.76)	0.045^*∗*^	0.19 (0.04–0.84)	0.029^*∗*^
Age (years)	0.99 (0.95–1.03)	0.726	0.99 (0.94–1.04)	0.652
Sex	0.53 (0.15–1.92)	0.333	1.87 (0.37–9.54)	0.452
LVEF (%)	0.98 (0.94–1.02)	0.289	1.02 (0.97–1.08)	0.439
Scr (mg/dL)	0.49 (0.11–2.14)	0.343	5.83 (0.32–107.78)	0.236
ALT (U/L)	0.98 (0.95–1.00)	0.083	0.98 (0.94–1.02)	0.314
Any DDI	1.27 (0.43–3.74)	0.667	0.81 (0.22–2.91)	0.744

^*∗*^Statistically significant (*P* < 0.05). ALT, alanine aminotransferase; DDIs, drug-drug interactions; CI, confidence interval; INR, international normalized ratio; LVEF, left ventricular ejection fraction; OR, odds ratio; PMWT, pharmacist-managed warfarin therapy; Scr, serum creatinine.

## Data Availability

The data used to support the findings of this study may be released upon request.

## Supplementary Materials

Supplementary Materials
*Table S1.* Initial dose titration protocol for inpatient warfarin users with different target INR.
