# Supplementary material for: The Impact of Pharmacist-Managed Service on Warfarin Therapy in Patients after Mechanical Valve Replacement
Source: Int J Clin Pract. 2022 Mar 17;2022:1617135. doi: 10.1155/2022/1617135 (PMC9159219; doi:10.1155/2022/1617135)
Supplement: Supplementary Materials — Table S1. Initial dose titration protocol for inpatient warfarin users with different target INR. [file 1617135.f1.docx]

Table S1 Initial dose titration protocol for inpatient warfarin users with different target INR

|  |  | | Increased sensitivity to warfarin* | General population |
| --- | --- | --- | --- | --- |
|  |  |  | Initial dose | |
|  |  |  | 1.0– 2.5 mg/day | 2.5–5.0 mg/day |
|  | Target INR | |  | |
|  | Low intensity 1.8–2.4 | Wide range 1.8–3.0 | Dose titration suggestions | |
| Day 2 | < 1.3 | < 1.3 | Maintain current dose | Maintain current dose |
|  | 1.3–1.7 | 1.3–1.7 | Maintain current dose or a decreased 20–50% dose | Maintain current dose or a decrease 20–50% dose |
|  | 1.8–2.1 | 1.8–2.5 | Decreased 50–75% dose | Decrease 20–50% dose |
|  | > 2.1 | > 2.5 | Discontinue | Discontinue |
| Day 3 | < 1.3 | < 1.3 | Maintain current dose or an increased 20–50% dose | Maintain current dose or an increased 20–50% dose |
|  | 1.3–1.7 | 1.3–1.7 | Maintain current dose | Maintain current dose |
|  | 1.8–2.4 | 1.8–3.0 | Maintain current dose or a decreased 20–50% dose | Maintain current dose or a decreased 5–20% dose |
|  | 2.5–3.0 | 3.1–3.5 | Discontinue or a decreased 50–75% dose | Discontinue or a decreased 20–50% dose |
|  | > 3.0 | > 3.5 | Discontinue | Discontinue |
| Day 4 | < 1.3 | < 1.3 | Increased 20–50% dose | Increase 50–75% dose |
|  | 1.3–1.7 | 1.3–1.7 | Maintain current dose or an increased 5–20% dose | Maintain current dose or an increased 5–20% dose |
|  | 1.8–2.4 | 1.8–3.0 | Maintain current dose or a decreased 5–20% dose | Maintain current dose or a decreased 5–20% dose |
|  | 2.5–3.0 | 3.1–3.5 | Decreased 20–50% dose | Decreased 20–50% dose |
|  | > 3.0 | > 3.5 | Discontinue | Discontinue |
| Day 5 | < 1.3 | < 1.3 | Increased 50–75% dose | Increased 50–100% dose |
|  | 1.3–1.7 | 1.3–1.7 | Maintain current dose or an increased 20–50% dose | Increased 20–50% dose |
|  | 1.8–2.4 | 1.8–3.0 | Maintain current dose | Maintain current dose |
|  | 2.5–3.0 | 3.1–3.5 | Decreased 5–20% dose | Decreased 5–20% dose |
|  | > 3.0 | > 3.5 | Discontinue | Discontinue |
| Day 6 | < 1.3 | < 1.3 | Increased 75–100% dose | Increased 100% dose |
|  | 1.3–1.7 | 1.3–1.7 | Increased 20–50% dose | Increased 50–100% dose |
|  | 1.8–2.4 | 1.8–3.0 | Maintain current dose | Maintain current dose |
|  | 2.5–3.0 | 3.1–3.5 | Decreased 5–20% dose | Decreased 5–20% dose |
|  | > 3.0 | > 3.5 | Discontinue | Discontinue |

* Patient characteristics for increased warfarin intensity: Age > 75 years, congestive heart failure, hyperthyroidism, end stage renal disease, febrile, impaired liver function, malignancy, CYP2C9 or VKORC1 polymorphism, post operation, decreased oral intake or feeding, malnutrition, hypoalbuminemia, diarrhea, drug-drug interactions, increased baseline INR

Abbreviations: VKORC1, vitamin K epoxide reductase complex.
